# Role of ethnicity in human papillomavirus vaccination uptake: a cross-sectional study of girls from ethnic minority groups attending London schools

**DOI:** 10.1136/bmjopen-2016-014527

**Published:** 2017-02-23

**Authors:** Lauren Rockliffe, Jo Waller, Laura A V Marlow, Alice S Forster

**Affiliations:** Department of Behavioural Science and Health, University College London (UCL), London, UK

**Keywords:** PREVENTIVE MEDICINE, PUBLIC HEALTH, ONCOLOGY

## Abstract

**Objectives:**

Research suggests that girls from ethnic minority groups are less likely to receive the human papillomavirus (HPV) vaccination than white British girls; however, the specific ethnic minority groups that have lower uptake have not been identified. This study aimed to examine the relationship between school-level uptake and ethnicity as well as uptake and other ethnicity-related factors, to understand which specific groups are less likely to receive the vaccination.

**Methods:**

Aggregated uptake rates from 195 schools were obtained for each of the three recommended vaccine doses from 2008 to 2010. Census data at the lower super output area (LSOA) level for the postcode of each school were also obtained, describing the ethnic breakdown of the resident population (ethnicity, language spoken, religion, proficiency in English and duration of residency in the UK). These were used as proxy measures of the ethnic make-up of the schools. The most prevalent non-majority group for each ethnicity and ethnicity-related factor was assigned to each school. Analyses explored differences in uptake by ethnicity and ethnicity-related factors.

**Results:**

No significant differences in vaccination uptake were found by ethnicity or ethnicity-related factors, although descriptive differences were apparent. Schools in areas where black ethnicities were the most prevalent non-white British ethnicities had consistently low rates of uptake for all doses. Schools in areas where some Asian ethnicities were the most prevalent non-white British ethnicities had consistently high rates of uptake for all doses. There was evidence of variability in mean uptake rates for ethnicities within ‘black’ and ‘Asian’ ethnic groups.

**Conclusions:**

Future research would benefit from focusing on specific ethnicities rather than broad ethnic categories. Replication of this study with a larger sample and using complete individual-level data, collected on a national level, would provide a clearer indication of where ethnic differences in HPV vaccination uptake exist.

**Strengths and limitations of this study**

- By examining the relationship between uptake of the human papillomavirus vaccination and ethnicity, and other factors related to ethnicity, we were able to identify ethnic groups for whom uptake of the vaccination was consistently low.
- We were able to demonstrate variability in mean uptake rates between ethnicities that would otherwise be grouped into broad ethnic categories, thus losing such detail.
- Further exploration is recommended with a larger sample size, using individual-level ethnicity and uptake data.

## Introduction

Human papillomavirus (HPV) is a common DNA virus that affects the skin and moist membranes that line parts of the body, such as the cervix. It is passed on through skin-to-skin contact and can be sexually transmitted. HPV infection is very common in sexually active individuals and there is a strong relationship between the number of lifetime and recent new sexual partner(s) and HPV infection.^1^ The majority of HPV infections are asymptomatic and transient; however, persistent infection with high-risk types of HPV is the primary cause of cervical cancer.^2^

A vaccination against HPV is available as part of the UK childhood immunisation schedule. The vaccination is offered free of charge, delivered mainly through schools and is recommended for all girls aged 12–13 years old. The vaccine is currently administered in a two-dose schedule, however, three doses were required prior to September 2014.^3^

Since the introduction of the vaccination, high coverage has been achieved in England with recent government figures reporting that 89.4% of girls received at least one dose in 2014/2015.^4^ However, research has shown that there are disparities in uptake between girls from white British backgrounds and ethnic minority backgrounds. Pilot implementation of the vaccination, carried out prior to national roll-out, concluded that although delivery was possible, non-white girls were less likely to be vaccinated than white girls^5^ and that uptake was significantly lower in schools with higher proportions of girls from ethnic minority groups.^6^ Comparable results have been reported following the introduction of the vaccination; Fisher *et al*^7^ examined routine vaccination data for 14 282 young women eligible for the HPV vaccination and found that girls from non-white British backgrounds (mixed ethnicity, Asian, black and ‘Chinese and other’) were less likely to initiate the vaccination compared with white British girls. Similar findings were also reported in a review of the literature which found that young black women were less likely to initiate the vaccination than young white women.^8^ It is important to note that not all studies in this review were UK based, which suggests that this is a global issue and not just UK specific.

When discussing ethnicity, it is important to consider other factors that might confound the relationship between ethnicity and health. Deprivation and ethnicity are often related^9^ and deprivation has been shown to predict uptake of the vaccination.^5^ However, the relationship between ethnicity and uptake of the HPV vaccination has been shown to be significant irrespective of deprivation^7^ and it has not been found to explain ethnic differences in acceptability^10^ or in uptake of the vaccination.^5^

Although differences in uptake rates between white British and ethnic minority groups have been established, the specific ethnic minority groups who are less likely to receive the vaccination have not been identified. Uptake of the vaccination has only been reported by broad ethnic categories, for example, ‘white’, ‘black’, ‘Asian’ or ‘other’. Many studies group participants in this way, however, such research is limited as minimal detail is provided as to the specific ethnicities included within each broad category. For example, in the UK, ‘Asian’ mainly denotes individuals from Bangladeshi, Indian and Pakistani backgrounds, but individuals from each of these groups are not homogenous. We are therefore unable to identify whether differences in uptake exist within ethnic minority populations and as a result are unable to target interventions towards specific groups.

The purpose of this study was to explore the relationship between school-level uptake of the HPV vaccination and ethnicity, as well as uptake and other factors related to ethnicity (eg, religion, language), to understand which specific ethnic minority groups are less likely to receive the HPV vaccination.

## Methods

### Note on terminology

Ethnicity has been defined as a ‘multifaceted quality that refers to the group to which people belong, and/or are perceived to belong, as a result of certain shared characteristics’.^11^ Bhopal^11^ states that ethnicity is different from race, nationality, religion and migrant status, but can include facets of these factors. Mirroring this, we use the term ‘ethnicity’ to refer to the construct of ‘ethnic group’ (eg, white British, African, Bangladeshi) and ‘ethnicity-related factors’ when referring to other factors relating to ethnicity (eg, religion, language, level of proficiency in English and duration of residence in the UK).

### Data

Aggregated HPV vaccination uptake rates were obtained for 195 London schools, for school years 2008/2009, 2009/2010 and 2010/2011 for each dose (prior to September 2014, three doses were administered), via personal contacts in borough immunisation teams. Aggregated data were used as complete individual-level data were not available.

Uptake data were matched with census data (2011) that were extracted from the Office for National Statistics website, for the lower super output area (LSOA) for the postcode of each school. The LSOA is a geographical area, comprising between 400 and 1200 households.^12^ We obtained data on ethnicity and four related factors: language spoken, religion, level of proficiency in English (can speak English very well; can speak English well; cannot speak English well and cannot speak English) and duration of residence in the UK (born in the UK; <2 years; 2 years or more but <5 years; 5 years or more but <10 years and 10 years or more).^13^ These data at the LSOA level were used as a proxy measure of the ethnic make-up of the school. We used census data to identify the most prevalent ethnicity, language spoken, religion, level of proficiency in English and duration of residence in the UK for each LSOA. Almost all of the schools' LSOAs comprised a resident population where the majority had the same ethnic characteristics as the majority of the UK population (ie, most residents were white British, spoke English as their main language, were Christian and were born in the UK). To allow us to explore how vaccination uptake varies by ethnicity and ethnicity-related factors (among those from minority groups), the most prevalent non-majority group^i^ within each ethnicity and ethnicity-related factor, for each LSOA, was assigned to each school^ii^ (eg, where white British was the most prevalent ethnicity, the second most common ethnicity in that LSOA was assigned to the school and where English was the most commonly spoken language in that LSOA, the second most commonly spoken language was assigned to the school). Where it was not possible to determine the most prevalent non-majority group (eg, where there were two non-majority groups of equal prevalence in a given LSOA), the data were recorded as missing.

### Statistical analysis

To maximise complete data, we computed one single uptake rate for each school, for each dose. We used 2010/2011 as the year of interest, but where data were missing, we replaced this with the rate for 2009/2010 and 2008/2009 if 2009/2010 data were missing.

Data were normally distributed, so one-way analyses of variance (ANOVAs) were conducted to examine school-level differences in uptake by area-level ethnicity and the four ethnicity-related factors for each of the three doses of the vaccine, for groups with counts >5. We performed a subanalysis to determine whether the relationship between ethnicity and uptake varied by whether each ethnicity represented a high or low proportion of the total resident population. Schools were grouped by whether the most prevalent non-white British ethnicity in their respective LSOA was a low or high proportion of the total population (using a median split). Those above the median (16.3%) were grouped as high and those below, as low. For each ethnicity we used Mann-Whitney U tests to examine differences in uptake, for all three doses of the vaccine separately, between schools categorised as ‘high’ and ‘low’ (excluding groups with counts <5).

## Results

Overall mean uptake of the vaccination across the 195 schools was 73.3% (SD; 16.6) for dose 1, 70.8% (SD; 17.7) for dose 2 and 66.2% (SD; 19.6) for dose 3. Uptake by ethnicity and other ethnicity-related factors is presented in table 1,^iii^ along with the results of the ANOVAs. No significant differences were found between ethnicity and uptake, language and uptake, religion and uptake or proficiency in English and uptake.

**Table 1:** Most prevalent non-majority ethnicity and ethnicity-related factors for each school and human papillomavirus (HPV) vaccination uptake

		Mean uptake					
	N (%)*	Dose 1% (SD)	p Value	Dose 2% (SD)	p Value	Dose 3% (SD)	p Value
Most prevalent non-white British ethnicity			0.172		0.195		0.274
Other white	115 (59.0)	71.4 (17.6)		69.3 (17.9)		64.8 (19.9)	
African	25 (12.8)	72.7 (19.7)		70.4 (21.3)		65.6 (24.8)	
Indian	20 (10.3)	77.3 (8.3)		75.4 (8.8)		72.1 (11.3)	
Bangladeshi	10 (5.1)	84.2 (8.9)		83.0 (10.1)		76.8 (9.1)	
Caribbean	12 (6.2)	72.5 (16.7)		67.7 (26.3)		60.8 (25.4)	
Other Asian	9 (4.6)	78.3 (12.1)		73.2 (9.4)		69.3 (11.7)	
Pakistani†	3 (1.5)	69.5 (10.4)		67.6 (11.3)		63.8 (14.6)	
Arab†	1 (0.5)	88.3		78.3		73.3	
Most prevalent non-English language‡			0.235		0.258		0.809
Other European	97 (50.5)	71.6 (17.5)		68.9 (19.1)		65.3 (19.9)	
South Asian	68 (35.4)	77.2 (13.3)		75.0 (14.3)		68.8 (18.9)	
East Asian	9 (4.7)	68.7 (13.3)		67.7 (12.4)		63.6 (10.7)	
Arabic	6 (3.1)	74.6 (12.4)		69.0 (10.4)		63.4 (13.7)	
Turkish	6 (3.1)	74.0 (31.8)		72.8 (31.5)		67.1 (34.4)	
African†	3 (1.6)	63.2 (26.4)		62.7 (25.8)		56.2 (34.3)	
West/Central Asian†	2 (1.0)	54.9 (31.7)		52.9 (30.7)		52.9 (30.7)	
French†	1 (0.5)	75.0		75.0		75.0	
Most prevalent non-Christian religion			0.099		0.204		0.531
No religion	140 (71.8)	74.0 (15.4)		71.6 (17.0)		66.9 (18.5)	
Muslim	43 (22.1)	73.1 (18.3)		70.2 (18.9)		66.0 (21.7)	
Jewish	9 (4.6)	61.7 (24.6)		60.8 (24.4)		59.2 (27.4)	
Hindu†	2 (1.0)	71.3 (6.5)		68.7 (2.8)		54.2 (16.0)	
Sikh†	1 (0.5)	83.3		81.7		78.3	
Most prevalent level of proficiency in English for non-native English speakers‡			0.587		0.877		0.804
Can speak English very well	140 (72.5)	73.9 (15.7)		71.2 (17.0)		66.2 (18.7)	
Can speak English well	53 (27.5)	72.5 (18.4)		70.7 (19.2)		67.0 (21.9)	
Most prevalent length of residence in the UK for non-UK born residents (years)†							
<2	2 (1.0)	86.7 (3.1)		75.9 (7.9)		74.1 (10.3)	
>5<10	1 (0.5)	55.6		55.6		37.0	
≥10	192 (98.5)	73.2 (16.6)		70.8 (17.7)		66.3 (19.6)	

*Number of schools providing data for at least one dose.

†Variables not included in the analysis of variance (ANOVA) due to small group sizes (<5).

‡N≠195 due to missing data.

Schools in areas where other white was the most prevalent non-white British ethnicity had the lowest rate of uptake for dose 1 (71.4%) and schools in areas where Caribbean was the most prevalent non-white British ethnicity had the lowest rates of uptake for doses 2 (67.7%) and 3 (60.8%). Overall, schools in areas where black ethnicities (African; Caribbean) were the most prevalent non-white British ethnicities had consistently low rates of uptake for all three doses. In comparison, schools in areas where some Asian ethnicities (Bangladeshi; Indian; other Asian) were the most prevalent non-white British ethnicities had consistently high rates of uptake for all three doses.^iv^

Schools in areas where the most commonly spoken non-English languages were East Asian, had the lowest rates of uptake for dose 1 (68.7%) and 2 (67.7%) and those in areas where Arabic was the most commonly spoken non-English language had the lowest rates for dose 3 (63.4%).

Schools in areas where Jewish was the most prevalent non-Christian religion, were found to have the lowest rates of uptake for all three doses of the vaccine (dose 1; 61.7%, dose 2; 60.8%, dose 3; 59.2%).

Schools in areas where English was spoken well by non-native English speakers, had the lowest rates of uptake for doses 1 (72.5%) and 2 (70.7%) and those in areas where English was spoken very well had the lowest rates of uptake for dose 3 (66.2%).

Variability in mean rates of uptake was apparent for ethnicities that would commonly be grouped into more broad ethnic categories. For example, for dose 1 mean uptake rates varied between schools in areas where Indian (77.3%, SD 8.3), Bangladeshi (84.2%, SD 8.9) and other Asian (78.3%, SD 12.1) were the most prevalent non-majority ethnicities, three ethnicities which are often grouped into a broad ‘Asian’ category. Similarly, mean uptake rates for dose 2 varied between schools in areas where the most prevalent non-majority ethnicities were African (70.4%, SD 21.3) and Caribbean (67.7%, SD 26.3), two ethnicities which are often grouped into a broad ‘black’ category.

The subanalyses showed that schools in areas where the most prevalent non-white ethnicity represented a low proportion of the total population had consistently higher uptake than schools in areas where the most prevalent non-white ethnicity was a high proportion of the total population. However, differences were not significant (see table 2).

**Table 2:** Human papillomavirus (HPV) vaccination uptake for schools where the most prevalent non-white British ethnicity was a low or high proportion of the total population

		Mean uptake					
	N (%)*	Dose 1% (SD)	p Value	Dose 2% (SD)	p Value	Dose 3% (SD)	p Value
African high	10 (5.1)	70.7 (21.8)	0.688	69.3 (21.3)	0.688	63.0 (25.6)	1.000
African low	15 (7.7)	74.0 (18.9)		71.2 (22.0)		67.2 (25.1)	
Indian high	5 (2.6)	81.6 (8.8)	0.303	80.7 (8.5)	0.303	74.2 (18.1)	0.303
Indian low	15 (7.7)	76.0 (8.0)		74.0 (8.4)		71.4 (8.7)	
Other white high	66 (33.8)	70.1 (20.5)	0.404	67.4 (21.0)	0.404	64.5 (20.9)	0.647
Other white low	49 (25.1)	73.1 (13.0)		72.0 (12.9)		65.3 (18.7)	
Caribbean high	2 (1.0)	61.7 (16.6)	†	61.2 (15.9)	†	50.9 (24.8)	†
Caribbean low	10 (5.1)	74.7 (16.8)		69.0 (28.3)		62.8 (26.4)	
Other Asian high	2 (1.0)	69.8 (17.2)	†	68.7 (17.6)	†	66.0 (20.3)	†
Other Asian low	7 (3.6)	80.7 (10.7)		74.7 (7.1)		70.3 (10.1)	
Pakistani high	2 (1.0)	74.3 (9.0)	†	71.5 (12.9)	†	67.6 (18.3)	†
Pakistani low	1 (0.5)	60.0		60.0		56.0	
Bangladeshi high	10 (5.1)	84.2 (8.9)	†	83.0 (10.1)	†	76.8 (9.1)	†
Bangladeshi low	0	–		–		–	
Arab high	0	–	†	–	†	–	†
Arab low	1 (0.5)	88.3		78.3		73.3	

*Number of schools providing data for at least one dose.

†Group sizes too small for test of difference to be run.

## Discussion

This study sought to identify whether differences existed in school-level uptake of the HPV vaccination by ethnicity or other ethnicity-related factors, in an attempt to understand which specific ethnic minority groups are less likely to receive the HPV vaccination. Comparisons were made between groups but no significant differences were found between ethnicity and uptake, or between any ethnicity-related factors and uptake, although descriptive differences were apparent.

There was evidence that uptake was consistently high among some Asian ethnicities (Bangladeshi; Indian and other Asian) and consistently low among black ethnicities (African and Caribbean). This supports previous research using individual-level data which found that uptake rates of the vaccination were lower for girls from black backgrounds than for girls from Asian backgrounds.^7^ There was also evidence of variability in mean uptake rates between ethnicities; differences were apparent between schools based in areas where Indian, Bangladeshi and Other Asian were the most common non-white British ethnicity and similarly between schools in areas where African and Caribbean were the most common non-white British ethnicity. These ethnicities are often grouped into the broad ethnic categories ‘Asian’ and ‘black’, respectively, resulting in a loss of detail about the individual ethnicities, which therefore limits what we know about their differences. These results demonstrate that there is variability between ethnicities when breaking down broad ethnic categories and suggests that further exploration is required.

Descriptively, the subanalysis showed that schools in areas where the most prevalent non-white ethnicity was a low proportion of the total population had higher uptake. The reason for conducting this subanalysis was to explore whether parents' vaccination choices are affected by the ethnic make-up of where they live (ie, ethnic minority parents living in areas where the majority of residents are white British may have higher uptake of the vaccination). Descriptively, this does appear to be the case; however, these differences were not significant and require further exploration with a larger sample in order to be able to draw such conclusions.

Knowledge and acceptance of the HPV vaccination has been shown to be lower in non-white groups,^14^ which may help to explain lower uptake rates. Religious beliefs and fear that the vaccination may encourage promiscuity have been cited as perceived barriers for parents from ethnic minority backgrounds.^10^ Furthermore, concerns about the age of vaccination and a lack of perceived risk regarding their daughter's susceptibility to infection have also been reported.^15^ Concerns and beliefs such as these provide some insight as to why vaccine acceptability and uptake are lower in ethnic minority groups, although further research will be required to identify variation between ethnicities that fall within the same broad ethnic categories.

The main limitation of this study is the use of ethnicity data collected at a LSOA level, rather than at an individual level. While proximity of residence to a school is usually a criterion for school entry in the UK, the data may not accurately reflect the characteristics of pupils in some schools. In addition, age-adjusted ethnicity was not available, so the ethnicity categories assigned to schools may not represent the school-aged resident population. There was some clustering of the schools within LSOAs and this was not taken into account when reporting mean uptake. No significant effects were detected in the analyses, which may be attributable to the area-level measure of ethnicity (and related factors), and/or a small sample size. Owing to this, power was only 0.47 for the effect size reported for the relationship between ethnicity and uptake. Based on our calculations, a sample of 372 schools would have been required to achieve sufficient power of 0.80.

The main strength of this study is the demonstration of variability between ethnicities that would normally be grouped into broad ethnic categories and the provision of further information about the specific ethnicities within the study that have lower uptake of the vaccination. These findings add detail to previous research in this area, although no conclusions can be drawn based on these results. What this study suggests to us however, is that policy recommendations should encourage the complete collection of ethnicity data at an individual level. This would provide us with a far more accurate indication about ethnicity and uptake rates and would not require the use of proxy measures, as used in the current study. Even where ethnicity is currently recorded at an individual level, it is incomplete.^16^ The complete collection of such data on a national level would allow us to develop a more thorough understanding of ethnic inequalities in uptake.

Going forward, further exploration is recommended with a larger sample size, using individual-level ethnicity and uptake data. Future research would benefit from focusing on specific ethnicities, rather than using broad ethnic categories to define participants. As this study has demonstrated, there is variability in mean uptake rates within broad ethnic groups. It is important to explore these differences so that we can gain a better understanding of the perceived barriers to vaccination that may exist for these specific groups rather than assuming all ethnicities grouped in any given broad ethnic category make the same decisions regarding HPV vaccination.

## Footnotes

i The majority group refers to the ethnic characteristics of the majority of UK residents, not the majority of residents in each LSOA (eg, white British, spoke English as their first language, Christian and born in the UK were always the majority groups).

ii Some groups were never the most prevalent non-majority group for any LSOA and so could not be included in the analysis (eg, ‘Buddhist’ was never the most prevalent non-Christian religion and the group ‘cannot speak English’ was never the most prevalent level of proficiency in English for non-native English speakers).

iii Uptake rates for groups with counts <5 are not described in the results section, although are tabulated for completeness.

iv Pakistani was not included due to small group size (<5).
